# Solving protein structure from sparse serial microcrystal diffraction data at a storage-ring synchrotron source

**DOI:** 10.1107/S205225251800903X

**Published:** 2018-07-20

**Authors:** Ti-Yen Lan, Jennifer L. Wierman, Mark W. Tate, Hugh T. Philipp, Jose M. Martin-Garcia, Lan Zhu, David Kissick, Petra Fromme, Robert F. Fischetti, Wei Liu, Veit Elser, Sol M. Gruner

**Affiliations:** a Laboratory of Atomic and Solid State Physics, Cornell University, Ithaca, NY 14853, USA; b Cornell High Energy Synchrotron Source (CHESS), Cornell University, Ithaca, NY 14853, USA; c Macromolecular Diffraction Facility at CHESS (MacCHESS), Cornell University, Ithaca, NY 14853, USA; d School of Molecular Sciences and Biodesign Center for Applied Structural Discovery, Biodesign Institute, Arizona State University, Tempe, AZ 85287, USA; e Advanced Photon Source, Argonne National Laboratory, 9700 South Cass Avenue, Lemont, IL 60439, USA; f Kavli Institute for Nanoscale Science, Cornell University, Ithaca, NY 14853, USA

**Keywords:** X-ray serial microcrystallography, sparse data, EMC algorithm, protein microcrystallography, storage-ring synchrotron sources

## Abstract

In recent years, the success of serial femtosecond crystallography and the paucity of beamtime at X-ray free-electron lasers have motivated the development of serial microcrystallography experiments at storage-ring synchrotron sources. However, especially at storage-ring sources, if a crystal is too small it will have suffered significant radiation damage before diffracting a sufficient number of X-rays into Bragg peaks for peak-indexing software to determine the crystal orientation. As a consequence, the data frames of small crystals often cannot be indexed and are discarded. Introduced here is a method based on the expand–maximize–compress (EMC) algorithm to solve protein structures, specifically from data frames for which indexing methods fail because too few X-rays are diffracted into Bragg peaks. The method is demonstrated on a real serial microcrystallography data set whose signals are too weak to be indexed by conventional methods. In spite of the daunting background scatter from the sample-delivery medium, it was still possible to solve the protein structure at 2.1 Å resolution. The ability of the EMC algorithm to analyze weak data frames will help to reduce sample consumption. It will also allow serial microcrystallography to be performed with crystals that are otherwise too small to be feasibly analyzed at storage-ring sources.

## Abstract 2

This work develops an analysis approach that can solve protein structures from data frames whose signals are too weak to be indexed. The approach is demonstrated on a serial microcrystallography data set collected at a storage-ring synchrotron source.

## 1. Introduction   

X-ray free-electron lasers (XFELs) have catalyzed several novel methods in biostructural science. Serial femtosecond crystallography (SFX), arguably the most successful of these methods so far, allows protein structure determination from nanocrystals by using X-ray pulses only femtoseconds long so as to outrun the damage process (Chapman *et al.*, 2011 ▸; Boutet *et al.*, 2012 ▸). Although developments in detector technology, sample delivery and data analysis have made SFX a viable technique, its wide use is limited by the scarcity of XFEL beamtime.

Despite the construction of XFELs worldwide, available beamtime in the near future will still be scarce compared with that provided by existing storage-ring synchrotron sources. This has inspired the development of serial microcrystallography experiments at current storage-ring sources (Gati *et al.*, 2014 ▸; Stellato *et al.*, 2014 ▸; Heymann *et al.*, 2014 ▸; Gruner & Lattman, 2015 ▸; Botha *et al.*, 2015 ▸; Nogly *et al.*, 2015 ▸; Roedig *et al.*, 2016 ▸; Martin-Garcia *et al.*, 2017 ▸). A serial microcrystallography experiment involves crystals sequentially delivered in random orientations into the X-ray beam. To merge the diffraction patterns, each frame must be indexed to determine the crystal orientation, which usually requires at least 20 to 30 resolvable Bragg peaks per frame. Since the pulse width of storage-ring sources is of the order of pico­seconds, radiation damage cannot be outrun in the same way as at XFELs. At storage rings the exposure time per crystal is limited by radiation damage. If the crystal is too small, too few X-rays to determine the crystal orientation will be diffracted prior to irreversible radiation damage. Therefore, serial crystallography at storage-ring sources has thus far relied on relatively large crystals. Frames with insufficient resolvable Bragg peaks for indexing, which we call ‘sparse frames’, are simply discarded. Proteins not bound up in large crystals are wasted for the purpose of structure determination.

Using the expand–maximize–compress (EMC) algorithm (Loh & Elser, 2009 ▸), we have developed an alternative analysis approach that makes use of the sparse frames. Unlike indexing algorithms that determine a definite orientation on a per frame basis, the EMC algorithm models the orientation of each frame probabilistically and reconstructs a consistent three-dimensional intensity model using all the data frames simultaneously. The information from a sparse frame still contributes to the reconstruction even though the frame alone cannot be indexed. This approach can reduce the usable crystal size in serial microcrystallography experiments at storage-ring sources and extract information from the sparse frames that would otherwise have been discarded.

This work is the latest contribution from a methodical programme to handle sparse frames. Philipp *et al.* (2012 ▸) and Ayyer *et al.* (2014 ▸) first showed that the probabilistic modeling of the EMC algorithm continues to hold even with just a few photons per frame in two- and three-dimensional shadowgraphy. Ayyer *et al.* (2015 ▸) subsequently applied the EMC algorithm to sparse frames collected from a small-molecule crystal rotated about a single axis, and Wierman *et al.* (2016 ▸) further extended the study to sparse frames taken from a large protein crystal rotated about a single axis. In order to sample a greater portion of the rotation space, Lan *et al.* (2017 ▸) analyzed sparse frames taken from a large protein crystal rotated about two orthogonal axes and developed computing schemes to speed up the reconstruction at high resolution.

Here, we describe a step-by-step analysis using the EMC algorithm on a real serial microcrystallography data set. Specifically, we threw away the strong crystal diffraction patterns and focused our analysis on the data frames that cannot be indexed by conventional means. In contrast with the Monte Carlo integration approach (Kirian *et al.*, 2010 ▸), our method uses the reconstructed crystal volumes, for all the data frames, when building the three-dimensional intensity model.

This paper is organized as follows: Section 2 describes the data set, the process of data reduction, and the modified version of the EMC algorithm used to address the individual crystal sizes and the large diffuse background scattering arising from the lipidic cubic phase (LCP) gel used to convey the crystals into the X-ray beam. Section 3 presents the results of the EMC reconstruction and the protein structure solution. In Section 4, we compare the experimentally measured background profile with the simulated scattering from water and discuss possibilities for background reduction. Additional technical details are presented in Appendices *A* [App appa] and *B* [App appb].

## 2. Materials and methods   

We tested our analysis method on a serial microcrystallography data set collected by Martin-Garcia *et al.* (2017 ▸) on the GM/CA 23-ID-D beamline at the Advanced Photon Source (APS). The raw data consist of 304 643 frames measured from hen egg white lysozyme microcrystals, ranging in size from 5 to 10 µm, at room temperature. We note that this data set is a representative subset of the data collected by Martin-Garcia *et al.* (2017 ▸) (364 724 frames in total), without any pre-selection. The crystals were sequentially delivered to the X-ray beam in random orientations by an LCP gel injector with a glass nozzle of 50 µm inner diameter (Weierstall *et al.*, 2014 ▸). The data were collected by a PILATUS3 6M detector with resolution of up to 1.75 Å in the detector corners. The detector has 2527 × 2463 square pixels, 172 × 172 µm each. In order to demonstrate the ability of our method to handle weak crystal diffraction data, we excluded data frames with more than 20 resolvable Bragg peaks, the empirical lower bound for normal indexing methods to succeed. In other words, we only considered the weak crystal diffraction patterns that were rejected from the structure determination by Martin-Garcia *et al.* (2017 ▸), which gives the 120 574 sparse frames used in our reconstruction.

### 2.1. Data reduction   

Our analysis started with identifying the frames containing crystal diffraction because the crystals were randomly distributed in the LCP gel. This process, also known as ‘hit finding’, first locates possible Bragg peaks from the diffuse background scatter. Our method is based on outlier detection. In the absence of crystal diffraction, the probability that a pixel measures a photon count, *K*, follows the Poisson distribution, *P_b_*(*K*) = exp(−*b*)*b* ^*K*^/*K*!, where *b* is an estimate (described below) of the photon number at that pixel due to the diffuse background scatter. Given *b*, we can identify an outlier pixel by its photon count being too large to be consistent with Poisson statistics. This consistency is defined *via* a photon count threshold, 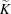, defined by the cumulative probability
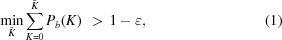
where ∊ is a small number that lets us set a false-positive rate (see below). If the photon count measured in the pixel exceeds the threshold 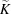, we assume that crystal diffraction contributed to the signal.

Since we had no prior knowledge of the background photon numbers *b*, we estimated them using the following self-consistent iterative scheme. Observing that the background scatter is generally azimuthally symmetric about the incident X-ray beam, we assumed that *b* only depends on the frame index *k* and the spatial frequency magnitude *q*. The initial values of *b_qk_* were obtained by averaging all photon counts in annular regions, after the pixel-wise correction of the polarization factor and solid angle. Because the number of pixels in these annular regions ranged from 10^3^ to 10^4^, the value of ∊ in equation (1) was set to 10^−5^ to reduce false positives arising from statistical fluctuations. In each iteration we used the current estimates of *b_qk_* to calculate the pixel-wise background estimates, *b_ik_*, by the relation 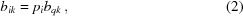
where *p_i_* is the product of the (positive) polarization factor and the solid angle of pixel *i*. From the values of *b_ik_*, we identified the outlier pixels and excluded them from the annular average for *b_qk_* in the next round. This procedure was repeated until the values of *b_qk_* converged, giving us a good estimate of the background scatter and a list of outlier pixels for each data frame.

**Figure 1:**
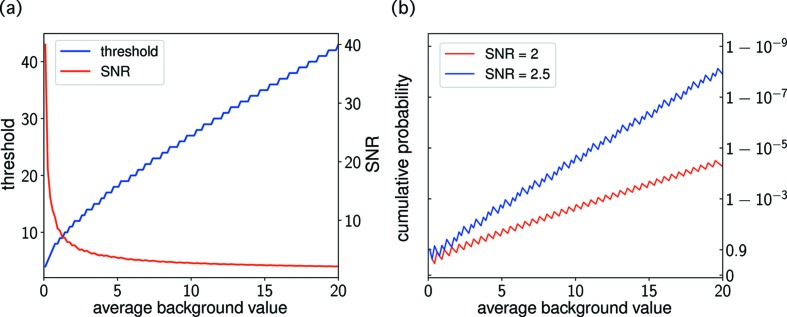
(*a*) The photon count thresholds determined by equation (1) with ∊ = 10^−5^. The SNR is defined as the ratio of the thresholds to the background estimates. (*b*) The cumulative probabilities *P*_*b*_(*K* 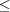 *b* × SNR) to measure a photon count *K* that is no larger than the thresholds *b* × SNR, defined by fixed values of SNR over a range of background estimates *b*.

The photon count thresholds 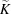, defined by equation (1) with ∊ = 10^−5^, are plotted in Fig. 1 ▸(*a*) over a range of background estimates *b*. Also shown is the signal-to-noise ratio (SNR), which is defined as the ratio of 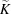 to *b*. We can see that the SNR takes on a wide range of values over *b*, especially when the values of *b* are close to zero. Since the background estimates in the data frames used in this study range from a fraction to 20 photons, the threshold values defined by the cumulative Poisson probability detects outliers in a more consistent way than those determined by a fixed SNR. Fig. 1 ▸(*b*) further illustrates this point by plotting the cumulative probabilities *P_b_*(*K* 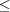 *b* × SNR) for different thresholds defined by fixed values of the SNR. Under this definition, photon counts greater than the threshold, *b* × SNR, are identified as outliers, which may result in many false positives at small values of *b*. In practice, the SNR is usually used along with other criteria that characterize a peak in the hit-finding process.

We defined a possible Bragg peak as a cluster with at least two but no more than ten contiguous outlier pixels, because most of the clusters have sizes smaller than five pixels. A cluster with more than ten contiguous outlier pixels was considered as originating from something other than a Bragg spot and was masked out for the rest of the analysis. As mentioned earlier, we discarded strong crystal diffraction patterns with more than 20 possible Bragg peaks. The possible Bragg-peak locations in the remaining data frames enabled us to estimate the lattice parameters by constructing a one-dimensional pseudo-powder pattern as follows: after mapping the possible peaks to reciprocal space, we recorded the distances between the centroids of the peaks in each data frame. By dividing the spatial frequency magnitudes into bins of the same size, the one-dimensional pseudo-powder pattern was given by a histogram recording the frequencies of the inter-peak distances in each bin. The inter-peak distances are a more reliable source of information about the lattice geometry than the distance from the center of the detector because of the beamstop. By assuming a primitive tetragonal lattice to simplify the analysis in this study, the lattice parameters were estimated by fitting the peaks in the one-dimensional pseudo-powder pattern.

In principle, we should be able to determine the lattice parameters from the one-dimensional pseudo-powder pattern even with no knowledge of the unit-cell type. This can be done by an exhaustive search over combinations of lattice parameters from unit cells with high symmetry to those with low symmetry. In challenging cases of crystals with low symmetry and large unit-cell dimensions, it may be necessary to take a separate diffraction measurement, that better resolves the inter-peak distances, with the detector further from the interaction point. The one-dimensional pseudo-powder pattern in this case would be the sum of resolvable peak values over spatial frequency magnitudes. Sample consumption should not be a concern here, since the number of peaks needed to populate the one-dimensional pseudo-powder pattern is of a similar order to the number of lattice parameters to be fitted (at most six). These low-resolution crystal diffraction patterns can also be incorporated into the EMC reconstruction to improve the statistics of Bragg intensities at low resolution.

Finally, we completed the hit-finding process by an exhaustive search in three-dimensional rotation space. The centroids of the possible peaks within a low-resolution cutoff in each frame were rotated over all rotation samples. We considered a frame to be a ‘crystal hit’ when at least three possible peaks matched the predicted Bragg positions within a predefined radius, *r*_p_, at some orientation, and all such orientations were recorded as the possible crystal orientations of this frame. This criterion reduced the number of frames by 60% for the later analysis and narrowed down the number of possible orientations for each frame. However, the possible orientations for each frame are still far from unique to orient the frames (see Section 3 for more details).

### 2.2. Model reconstruction   

#### 2.2.1. Signal model   

The diffraction pattern of each crystal hit can be modeled as the Poisson sample from the incoherent sum of the crystal diffraction and the background estimates, *i.e.* the average photon number due to the diffuse background scatter. Consider data frame *k* that records the diffraction of a crystal at orientation *j*. The average photon number 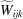 measured by pixel *i* is given by 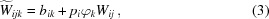
where φ_*k*_ is a scale factor proportional to the crystal volume, the X-ray beam fluence and the travel time of the crystal across the beam, and *W_ij_* denotes the value sampled by pixel *i* from the three-dimensional crystal intensity model *W* at crystal orientation *j*. In this study, all crystal volumes refer to the portion of crystals illuminated by the X-ray beam over the exposure time of a data frame. The Poisson sample from 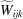 gives the photon count *K_ik_* with the crystal orientation unmeasured. Our main task in this study is to reconstruct *W* and φ_*k*_ given the data *K_ik_* and background estimates *b_ik_*.

#### 2.2.2. EMC algorithm   

We reconstructed the models *W* and φ using the EMC algorithm (Loh & Elser, 2009 ▸), which iteratively updates the current models by maximizing the data likelihood. Each iteration of the EMC algorithm consists of three steps: expand (E), maximize (M) and compress (C). The E step calculates the tomograms *W_ij_* from the current three-dimensional intensity model *W*(**p**) by linear interpolation 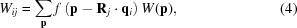
where *f*(·) is the interpolation weight, **p** denotes the three-dimensional grid points in reciprocal space, **R**_*j*_ is the rotation matrix that brings the laboratory frame to the crystal reference frame when the crystal has orientation *j*, and **q**_*i*_ is the spatial frequency of pixel *i* in the laboratory frame. We adopt the convention |**q**| = 2sin(θ/2)/λ, where θ is the scattering angle and λ represents the X-ray wavelength.

The M step updates the models by maximizing an expected log-likelihood function 
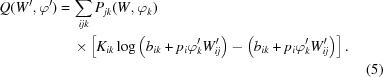
Here, *P_jk_*(*W*, φ_*k*_) denotes the conditional probability that data frame *k* records the diffraction of a crystal at orientation *j* given the current models: 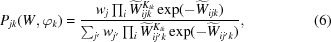
where *w_j_* is the fraction of the continuous rotation group assigned to rotation sample *j*. However, simultaneous updates for *W*′ and φ′ are nontrivial because they appear as products in *Q*. As suggested by Loh *et al.* (2010 ▸), the models are instead updated by maximizing *Q* with one or other of these parameters, *W*′ or φ′, held fixed in each EMC iteration. This alternating update rule converts the original problem into two sets of minimizations 
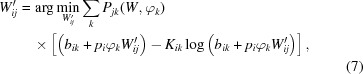


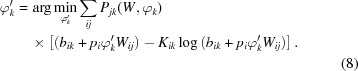
Since the functions to be minimized in equations (7) and (8) are convex, the minima can be readily found by a line search, *i.e.* a simple numerical algorithm to locate minima in one dimension (Press *et al.*, 2007 ▸). We imposed the non-negativity constraint on 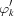 when solving equation (8) to prohibit negative crystal volume. On the other hand, negative values of 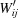 are allowed when solving equation (7), as a result of noise.

The C step enforces consistency between different tomograms 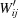 by merging them to form a new three-dimensional intensity model, *W*′. If the updated model is φ′ in an iteration, the C step is skipped and the current model, φ, is replaced by φ′ to start the next iteration. The tomograms 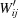 are mapped to the updated three-dimensional intensity model, *W*′(**p**), by 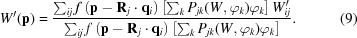
The tomograms 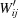 are weighted by 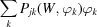 to reflect the frequency of orientation *j* populated by the data frames with a weight corresponding to the signal strength of the frame. The construction of *W*′ completes the C step and the iterations continue until the models converge: *W* ≃ *W*′ and φ ≃ φ′.

## 3. Results   

### 3.1. Background estimate and hit finding   

**Figure 2:**
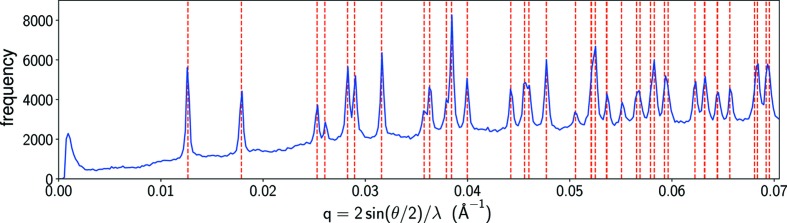
The one-dimensional pseudo-powder pattern generated from the frequency of the inter-peak distances in reciprocal space. Red dashed lines indicate peaks predicted by a primitive tetragonal lattice with lattice parameters *a* = 79.1 and *c* = 38.4 Å. The peak closest to the origin represents pairs of Bragg-peak candidates that are very close to each other. These pairs are actually fragments of Bragg spots of a larger size.

Using the method described in Section 2.1, we estimated the pixel-wise background estimates *b_ik_* and identified the outlier pixels. Bragg-peak candidates were identified by two to ten contiguous outlier pixels and clusters larger than this size were masked out. Data frames with more than 20 candidate peaks were discarded to show that the EMC algorithm is able to reconstruct the three-dimensional crystal intensity from the sparse data frames, where normal indexing methods, including the one used by Martin-Garcia *et al.* (2017 ▸), would fail. Using the remaining data frames, we calculated the inter-peak distances in reciprocal space to generate the one-dimensional pseudo-powder pattern (Fig. 2 ▸). The lattice parameters were estimated as *a* = 79.1 and *c* = 38.4 Å assuming a primitive tetragonal lattice.

**Figure 3:**
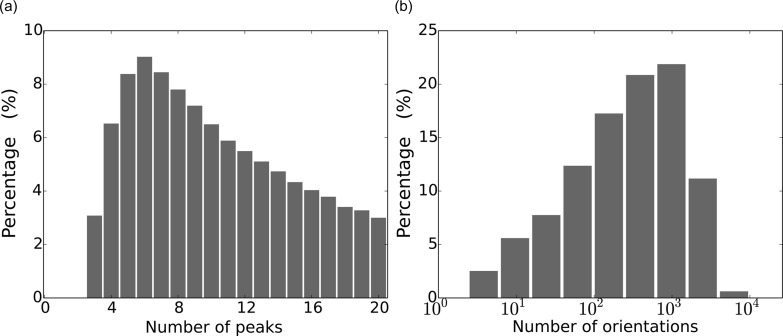
(*a*) The number of possible peaks in each crystal-hit frame. Data frames with more than 20 peaks were excluded from this study. (*b*) The number of possible orientations for each crystal-hit frame, determined by an exhaustive search of rotation space using the identified peaks within 4 Å.

We later rotated the candidate peaks within 4 Å resolution in each frame over all rotation samples to find the possible crystal orientations, where at least three peaks match the Bragg positions predicted by the lattice parameters. Data frames with no such orientations were discarded. Rotations were sampled by the 600-cell subdivision method at order *n* = 70 (Loh & Elser, 2009 ▸), which corresponds to an angular resolution of 0.944/*n* ≃ 13.5 mrad. This procedure reduced the data to 120 574 crystal-hit frames, with the statistics shown in Fig. 3 ▸. We note that, in general, a given crystal can be in any orientation. Practically speaking, discretization of all possible orientations results in hundreds to thousands of possibilities as a consequence of two factors: (i) the large angular size of low-resolution peaks, given that high-resolution peaks may not be resolvable due to their weak signals, and (ii) the inclusion of peak candidates arising from multiple crystals or any source of scatter other than protein crystals. The EMC algorithm addresses these two issues by making use of all the available photon count values.

### 3.2. EMC reconstruction   

#### 3.2.1. Low-resolution reconstruction   

We began with a low-resolution reconstruction because the computation time of the EMC algorithm is proportional to the number of pixels and the number of rotation samples. Pixels with a resolution higher than 4 Å were masked out in the 120 574 selected frames, and the rotation samples for each frame were limited to the possible crystal orientations recorded in the hit-finding process. All photon counts within the resolution cutoff were input to the EMC algorithm to reconstruct both the strong and weak intensities. We seeded the three-dimensional intensity model *W* with three-dimensional Gaussians of random height at each Bragg position, and only allowed the voxels within the predefined radius *r*_p_ about the Bragg positions to be non-zero throughout the reconstruction. The scale factors φ_*k*_ were initialized by the average value of the identified peaks in each frame. To achieve the highest resolution, we imposed tetragonal and Friedel symmetries on the values of *W* after each update to increase the SNR of the Bragg peaks. We note that EMC reconstructions normally succeed even without imposing symmetry (Wierman *et al.*, 2016 ▸; Lan *et al.*, 2017 ▸).

To rapidly obtain a rough estimate of *W*, we fixed the values of φ_*k*_ and only updated *W* in the first few iterations. Subsequently, we alternated the updates between *W* and φ until the models converged. Depending on the crystal concentration in the sample-delivery medium, a data frame may record diffraction signals from multiple crystals. Since our algorithm assumes that each crystal-hit frame only contains a single crystal, we had to reject multi-crystal frames to avoid compromising the reconstruction. This task was completed using the converged probability distribution *P_jk_*. When a data frame has non-negligible probabilities at two independent orientations *j*_1_ and *j*_2_, which cannot be related by the crystal point-group symmetry, it is likely that the diffraction signals were scattered from two different crystals. With probabilities greater than 0.05 considered non-negligible, a data frame has 1.02 independent orientations on average. We identified 528 multi-crystal frames and excluded them, together with the 2142 frames with φ_*k*_ = 0, from the later analysis. Using the remaining 117 904 single-crystal frames, we updated *W* for a few more iterations by fixing the values of φ_*k*_ until the new convergence was reached.

**Figure 4:**
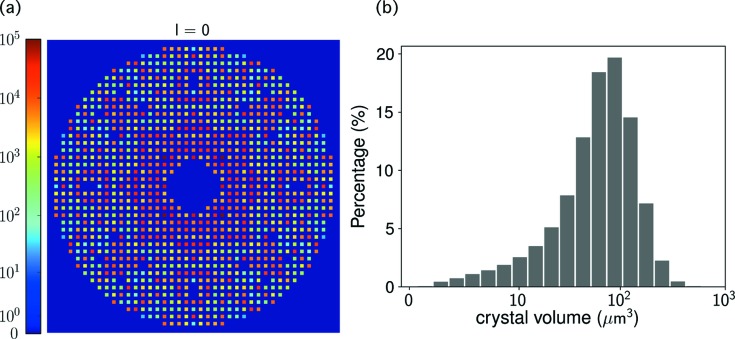
(*a*) The central slice of the low-resolution three-dimensional intensity model, *W*, perpendicular to the *l* axis of the crystal. Each spot represents an integrated Bragg peak in arbitrary units, with the negative reflections thresholded to zero for rendering. (*b*) The reconstructed crystal-volume distribution for the single-crystal frames. The values of the crystal volume were rescaled so that the largest crystal size is 10 µm.

Fig. 4 ▸(*a*) shows the central slice of the reconstructed three-dimensional intensity model, *W*, perpendicular to the *l* axis of the crystal. Each spot represents the integrated value of a Bragg peak in arbitrary units. After dividing the reconstructed values of φ_*k*_ by the beam fluence and the crystal exposure time, we obtained crystal-volume estimates for the single-crystal frames. In order to put these on an absolute scale, we further rescaled their values so that the largest crystal has a size of 10 µm, the value reported by Martin-Garcia *et al.* (2017 ▸). The resulting crystal-volume distribution has 73% of the frames with a crystal volume below 100 µm^3^ (Fig. 4 ▸ *b*). Since our analysis excluded frames with more than 20 peaks, which generally have larger crystal sizes, this distribution represents the upper limit of the crystal volume illuminated by the X-ray beam.

#### 3.2.2. High-resolution reconstruction   

**Figure 5:**
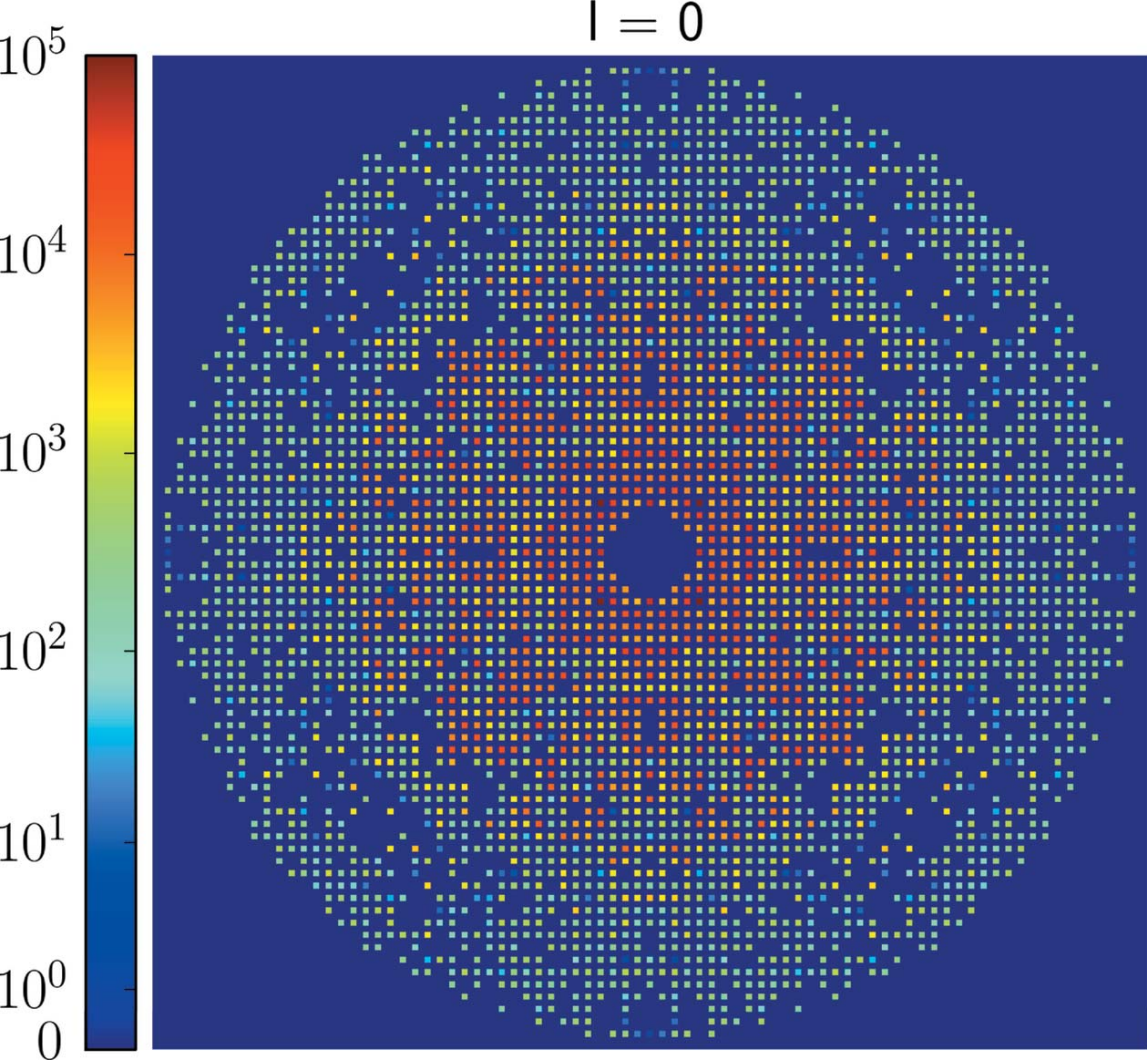
The central slice of the high-resolution three-dimensional intensity model, *W*, perpendicular to the *l* axis of the crystal, on the same scale as Fig. 4 ▸(*a*). Negative reflections were thresholded to zero for rendering.

Based on the low-resolution models, we extended our reconstruction to high resolution using data up to 2 Å. We initialized the three-dimensional intensity model *W* by three-dimensional Gaussians of random height at each Bragg position, and replaced the voxel values within 4 Å resolution with the low-resolution three-dimensional intensity model. To reduce the computation time for the high-resolution reconstruction, we implemented the local update scheme of the EMC algorithm. This scheme limits the rotation samples searched for each data frame to those neighboring the orientations that were given a non-negligible probability in the low-resolution reconstruction (Lan *et al.*, 2017 ▸). Here the orientation sampling was set at order *n* = 140, which corresponds to an angular resolution of 6.7 mrad. The update was limited to the three-dimensional intensity model *W*, because we believe the values of φ_*k*_ are reliably determined by the low-resolution peaks. Tetragonal and Friedel symmetries were imposed after each update of *W* to increase the SNR of the Bragg peaks. Fig. 5 ▸ shows the central slice of *W* perpendicular to the *l* axis of the crystal, on the same scale as Fig. 4 ▸(*a*). The uncertainties of the integrated intensities were estimated following the procedure described in Appendix *A* [App appa].

**Figure 6:**
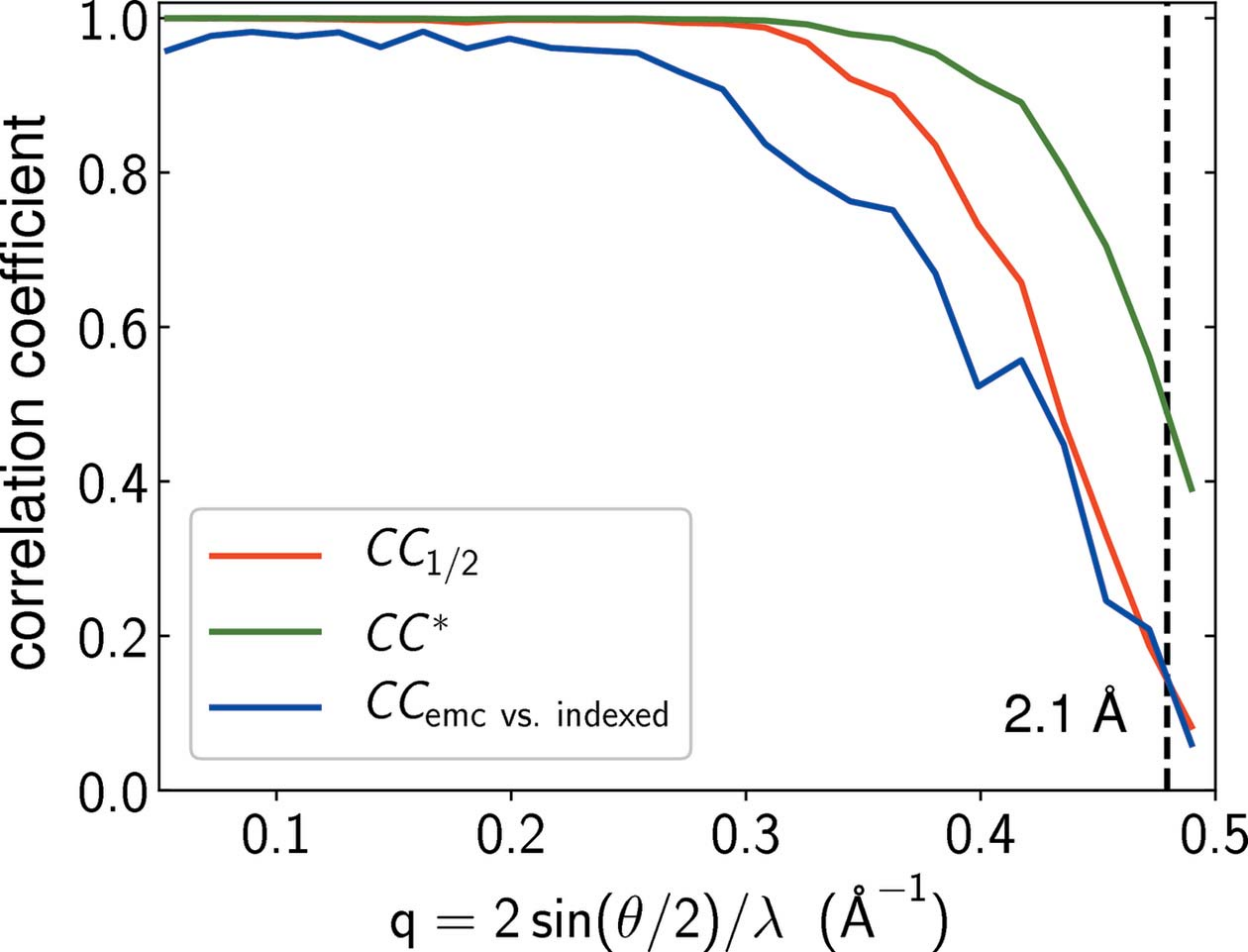
The correlation coefficients that validate the quality of our reconstruction. The values of CC_1/2_ show the correlation between Bragg intensities reconstructed independently from two halves of the data frames. Using equation (10), the values of CC*, the correlation coefficient between reconstructed intensities and the underlying true signals, are estimated from the values of CC_1/2_. The other correlation coefficient, CC_emc vs. indexed_, measures the consistency between our reconstructed intensities and those obtained by Martin-Garcia *et al.* (2017 ▸) from the indexed frames.

We evaluated the reproducibility of the reconstruction using CC_1/2_, the correlation coefficient between two sets of Bragg intensities reconstructed independently from two halves of the data frames. The values of CC_1/2_ were calculated as follows. The 117 904 single-crystal frames were separated into two halves, from which we carried out two independent reconstructions. The reciprocal space was then divided into shells with equal spacing, and the correlation coefficients CC_1/2_ were computed between the unique reflections from the two reconstructions in each shell. As shown in Fig. 6 ▸, the positive values of CC_1/2_ throughout the spatial frequency magnitudes validate the reproducibility of our approach. The values of CC_1/2_ can be further used to estimate another correlation coefficient, CC*, through the relation 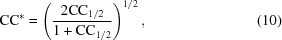
where CC* measures the correlation between the reconstructed intensities and the underlying true signals (Karplus & Diederichs, 2012 ▸). The resolution of the reconstruction is conventionally determined at the value where CC* drops to 0.5, which corresponds to 2.1 Å in our case.

A more direct validation of our reconstruction comes from the comparison of our reconstructed intensities with those calculated from the indexed peaks using the Monte Carlo integration approach by Martin-Garcia *et al.* (2017 ▸). Dividing the reciprocal space into shells of equal spacing, we calculated the correlation coefficient between the unique peaks from the two sets of Bragg intensities in each shell. Also shown in Fig. 6 ▸, the correlation coefficient stays well above zero until the resolution cutoff of 2.1 Å, which demonstrates the consistency between the Bragg intensities solved from the two different approaches. When the indexed peaks sufficiently sample crystals of various shapes, sizes and orientations, the Bragg intensities computed by the Monte Carlo method would in principle correspond to the true signals. In that case, the curve of the correlation coefficient calculated here should move towards the curve of CC* in Fig. 6 ▸.

### 3.3. Model building, refinement and structure solution   

Model-building and refinement steps were carried out in a manner similar to those performed by Martin-Garcia *et al.* (2017 ▸), with the intent of validating the EMC approach by a direct comparison with the structure solved from the indexed frames, PDB entry 5uvj. The French–Wilson correction (French & Wilson, 1978 ▸) was executed to estimate the structure-factor magnitudes from the reconstructed weak or negative Bragg intensities. The phases of the structure factors were built from the same template as used by Martin-Garcia *et al.* (2017 ▸), PDB entry 4zix (Fromme *et al.*, 2015 ▸), using mol­ecular replacement with *MOLREP* (Vagin & Teplyakov, 2010 ▸).

The structure solution was then iteratively refined and inspected using *REFMAC*5 (Kovalevskiy *et al.*, 2018 ▸) in the *CCP*4 suite (Potterton *et al.*, 2018 ▸) and *Coot* (Emsley & Cowtan, 2004 ▸), respectively. The structure was refined to 2.1 Å resolution, with *R*_work_/*R*_free_ of 22.2%/28.2%, an average *B* value of 39.8 Å^2^, and root-mean-square deviations (r.m.s.d.s) for bonds and angles of 0.013 Å and 1.21°, respectively. Most of the side-chain conformations were determined exactly, though some solvent-exposed side chains show multiple conformations. A sodium atom was added, as judged by the electron density within the known octahedral coordination of the four residues of the sodium ion (see also Fig. 9). The refinement statistics for the EMC-reconstructed structure solution and the structure solved by Martin-Garcia *et al.* (2017 ▸) are summarized in Table 1 ▸ for comparison.

### 3.4. Structural comparison with PDB entry 5uvj   

**Figure 7:**
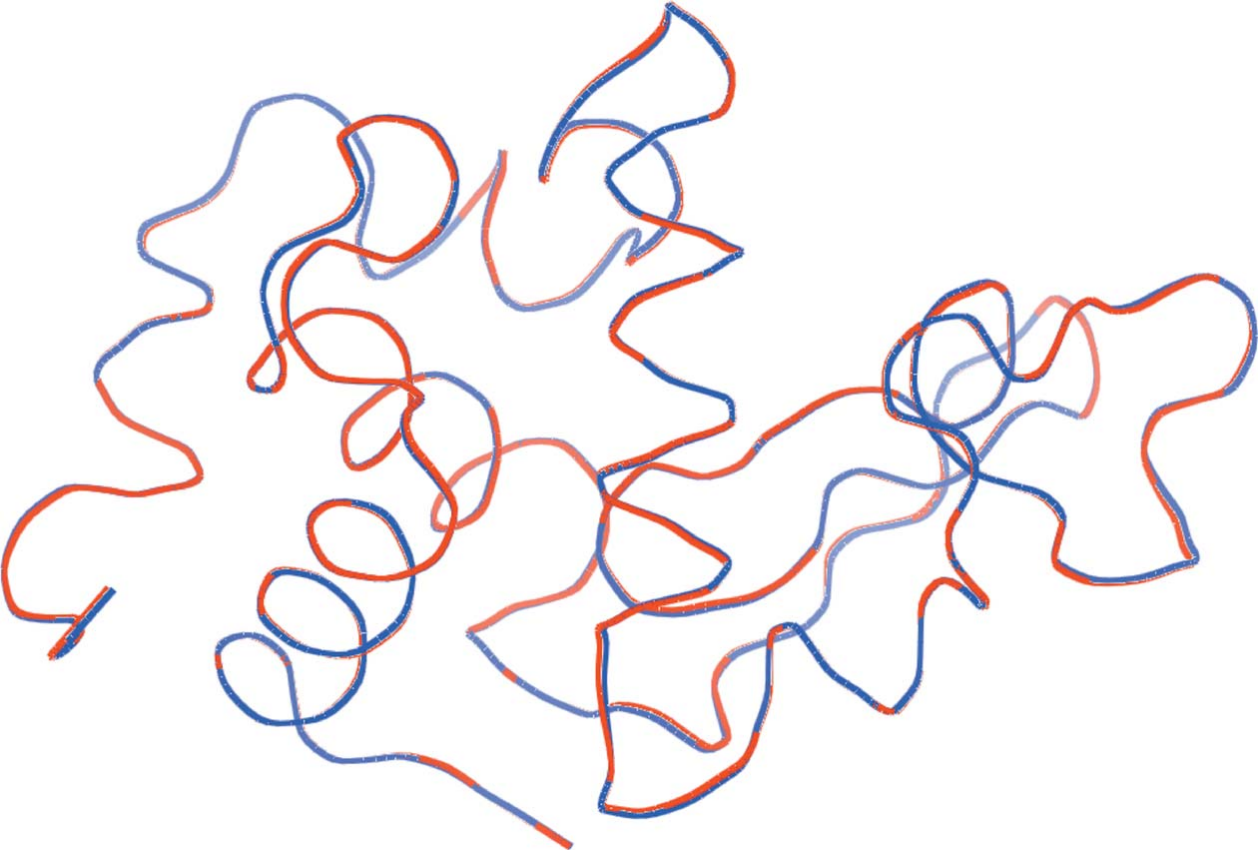
Superposition of the ribbon representations of the backbone chains of our structure solution (blue) and the structure of 5uvj (red) solved by Martin-Garcia *et al.* (2017 ▸), showing insignificant differences in structure. The C_α_ atoms between the two structures have an r.m.s.d. of 0.131 Å. Deviations greater than this occur mostly in the solvent-exposed regions, with a maximum deviation of 0.337 Å, though the deviations are only apparent by occasional changes in color from red to blue along the backbone.

**Figure 8:**
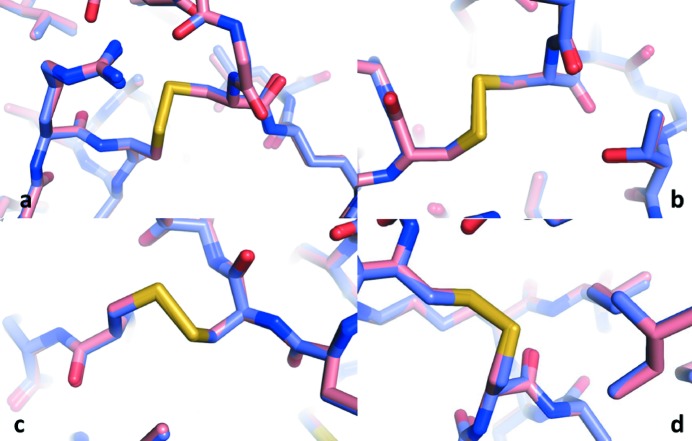
Superpositions of the four disulfide bonds (yellow) between our structure solution (light red) and the structure of 5uvj (light blue) solved by Martin-Garcia *et al.* (2017 ▸). (*a*) Cys6–Cys127, (*b*) Cys30–Cys115, (*c*) Cys64–Cys80 and (*d*) Cys76–Cys94. The average deviation for the atoms of the thiol groups is 0.12 Å. Changes are mostly insignificant, and only apparent in splits from light red to light blue.

**Figure 9:**
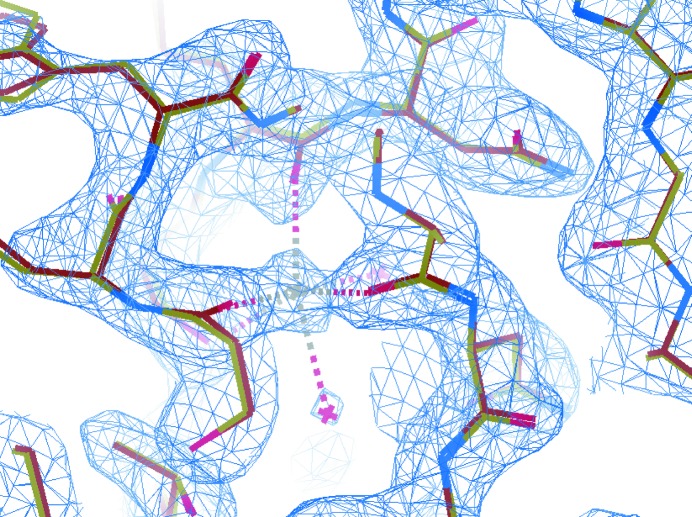
The 2*F*_o_ − *F*_c_ electron-density map (blue) contoured around the sodium-ion binding pocket, where *F*_o_ represents the observed structure-factor magnitudes, and both *F*_c_ and the phases were calculated from the initial model for phasing (PDB entry 4zix). Also shown is the alignment of our structure solution (yellow) and the structure of 5uvj (red) solved by Martin-Garcia *et al.* (2017 ▸). Small deviations are seen more clearly between the structures near the solvent-exposed regions in the yellow and red representations. Waters are seen as red crosses, the sodium ion as a gray cross, and the residues coordinating the sodium atom (Ser60, Cys64, Arg71 and Ser72) as red dashes. The oxygen atoms (in red) seen near the top of the figure have the largest displacement of 0.13 Å among all the atoms shown.

In this section, we compare our structure solution with the structure solved from the indexed frames by Martin-Garcia *et al.* (2017 ▸; PDB entry 5uvj). The electron-density maps of the structures were analyzed and rendered using *PyMOL* (Schrödinger LLC, 2015 ▸). Fig. 7 ▸ shows ribbon representations of the backbone chains of our molecular model (blue) and the structure of 5uvj (red). The C_α_ atoms between the two structures have an r.m.s.d. of 0.131 Å, which is visible as an occasional change between the red and blue colors along the backbone chain. Deviations greater than this value occur mostly in the solvent-exposed regions, with a maximum deviation of 0.337 Å. The r.m.s.d. value for the entire protein molecule between the two structures is 0.138 Å, with a maximum deviation of 0.338 Å. More specifically, Fig. 8 ▸ displays the disulfide bonds (yellow) within two superimposed structures, the EMC-reconstructed one (light red) and that of PDB entry 5uvj (light blue), showing insignificant deviations between the structures within the more radiation-damage-prone bonds. The average deviation for the atoms of the thiol groups is 0.12 Å. Fig. 9 ▸ shows the 2*F*_o_ − *F*_c_ electron-density map in blue mesh, where *F*_o_ represents the observed structure-factor magnitudes, and both *F*_c_ and the phases were calculated from the initial model for phasing, PDB entry 4zix. Also shown is the superposition of our structure solution (yellow) and that of PDB entry 5uvj (red) around the sodium-ion binding pocket. The largest discrepancy in atomic displacements (with a deviation up to 0.33 Å) comes from the solvent-exposed side chains.

## 4. Discussion   

**Figure 10:**
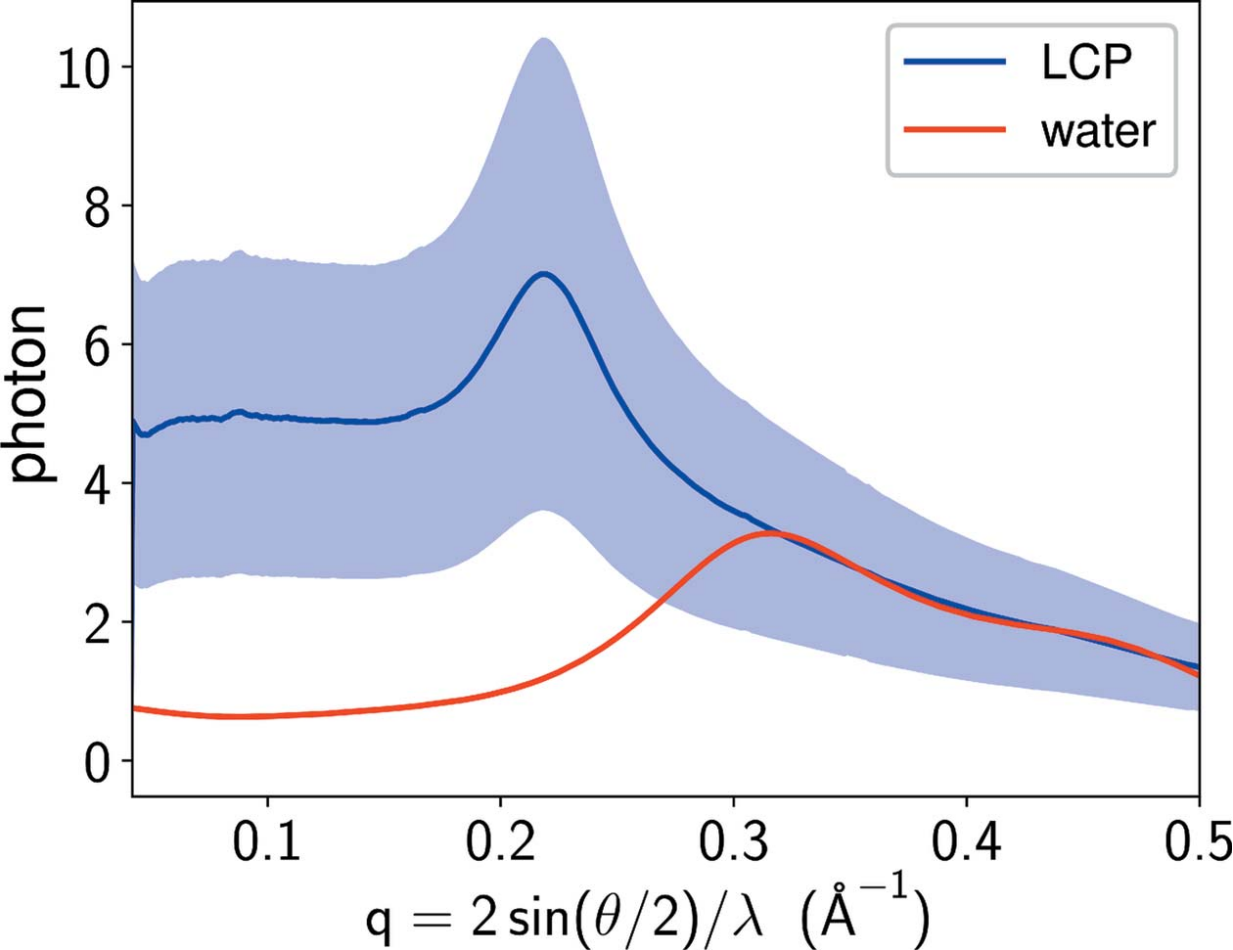
The scattering profiles of LCP and water, which were generated by the weighted average of the background estimates obtained in Section 2.1 and simulation, respectively. The shaded region is within one standard deviation of the average scattering profile of LCP. The large standard deviation is mainly caused by jittering of the LCP stream.

The major source of error that limits the quality of our reconstruction is the high background scatter from the LCP gel. Here the error refers to the statistical error arising from background intensity fluctuations, which becomes substantial and severe for weak reflections. From the estimated X-ray beam size (different beam sizes of 5, 10 or 20 µm were used at different times during the data collection), the diameter of the LCP gel column (50 µm) and the reconstructed crystal volumes (Fig. 4 ▸ *b*), we can estimate the total number of photons scattered by LCP to be tens to thousands of times more than that scattered by the crystal in each data frame. In Fig. 10 ▸, we compare the scattering profiles of LCP and water. The scattering profile of LCP was estimated by the average of the azimuthally symmetric background obtained in Section 2.1. Since the X-ray beam size and detector exposure time were varied in different periods of beamtime, the background signals in each frame were rescaled before the average to have a nominal beam size of 10 µm and a detector exposure time of 0.1 s. Under the same experimental conditions, we simulated the scattering profile from a water column of 50 µm diameter using the experimentally measured pair-distribution function (Narten & Levy, 1971 ▸; Skinner *et al.*, 2013 ▸). In contrast with water, LCP scatters a large number of photons within 3 Å resolution.

The high background scattering from LCP has motivated a search for sample-delivery media that scatter fewer background photons. For example, Conrad *et al.* (2015 ▸) used agarose to reduce background scattering, although the agarose stream tends to be unstable under ambient pressure. On the other hand, the sodium carboxymethyl cellulose (NaCMC) and poly(ethylene oxide) (PEO) reported by Kovácsová *et al.* (2017 ▸) and Martin-Garcia *et al.* (2017 ▸), respectively, produce stable streams and lower background scattering than LCP, and therefore may be good substitutes for LCP. Another option for background reduction is to use the fixed-target approach. As demonstrated recently by Roedig *et al.* (2016 ▸) and Owen *et al.* (2017 ▸), rapid data collection can be achieved by fast scanning through micro-patterned silicon chips mounted with protein microcrystals. Nevertheless, the challenge of the chip methods is to avoid preferential crystal orientations. Other possible methods include microcrystal droplets deposited on low-background tape carriers (Fuller *et al.*, 2017 ▸).

The structure solved by the EMC approach using sparse frames compares very well with the structure solved by Martin-Garcia *et al.* (2017 ▸) using indexed frames. Small discrepancies in atomic positions between the two structures reside mainly on the solvent-exposed side chains, and can be attributed to multiple conformers. The higher average *B* value of our structure suggests that the data frames we used may have come from less ordered and possibly more weakly diffracting crystals, which are exactly the features we expect from sparse frames.

The ability to analyze sparse crystal diffraction data allows the use of very small or weakly diffracting protein crystals at storage-ring synchrotron sources. In order to keep these crystals within the safe radiation dose, the resulting diffraction patterns usually contain insufficient photons for the normal indexing methods to succeed. From our previous proof-of-concept studies, reconstruction is feasible for crystal sizes as small as 1–2 µm within a tolerable radiation dose, given sufficient reduction of background scattering (Wierman *et al.*, 2016 ▸; Lan *et al.*, 2017 ▸). The successful application of the EMC algorithm to data collected from such small crystals will be a great advance in protein structure determination at storage-ring sources, and at the same time will ease the high demands for XFEL beamtime. An extension to include polychromatic data, where only 1% of the frames are needed due to the 100-fold increase in X-ray energy bandwidth, could dramatically reduce the amount of sample needed as well as the computation time. Continued development of lower-background microcrystal carrier methods would facilitate the application of our method.

Extracting weak signals from diffuse background scattering is not a task just limited to serial microcrystallography. When crystals are disordered, continuous diffraction of the protein molecules arises between the Bragg peaks (Ayyer *et al.*, 2016 ▸; Meisburger *et al.*, 2017 ▸). Separating this continuous diffraction from background scattering becomes nontrivial when the signals are Poisson-limited. The analysis scheme recently developed by Chapman *et al.* (2017 ▸) subtracts the azimuthally symmetric background from the diffraction signal using the ‘noisy Wilson distribution’. It would be interesting to adapt the EMC algorithm to this noisy Wilson distribution to analyze unindexable diffraction patterns collected from disordered crystals. Another application lies in single-particle imaging (SPI), where each measurement is composed of the continuous diffraction of a randomly oriented bioparticle superimposed on background noise. If the statistical model for the intensity distribution in SPI is known, this information can be incorporated into the EMC algorithm to reconstruct simultaneously the three-dimensional intensity of the bioparticle and the initially unknown background.

## 5. Conclusion   

In this study, we have developed an approach to analyze a serial microcrystallography data set whose signals are too noisy to be considered by the prior state of the art. In particular, weak crystal diffraction signals can be extracted from diffuse background scattering to form a three-dimensional intensity volume. This approach reduces sample consumption by making use of all the available data frames. We have demonstrated that a protein structure can be solved from the data frames that are discarded by the current analysis workflow. The partial reflections are assembled by rescaling the crystal diffraction signals in each data frame with the reconstructed crystal volumes. The reconstruction of the crystal-volume distribution may also be useful for the development of sample-injection technology.

The source code for the EMC analysis approach is available at https://github.com/tl578/EMC-for-SMX under the terms of version 3 of the GNU General Public License (GPLv3). A tutorial on the implementation details of the code can be found at https://github.com/tl578/EMC-for-SMX/wiki.

## Figures and Tables

**Table 1:** The refinement statistics of our structure solution and the structure solved by Martin-Garcia *et al.* (2017 ▸) (PDB entry 5uvj)

	EMC	5uvj
Resolution (Å)	22.52–2.10	35.00–2.05
Reflections	7417	7164
Atoms	1019	1023
Protein atoms	1002	1002
Water, ligands and ions	17	21
*R*_work_/*R*_free_ (%)	22.2/28.2	22.8/26.8
R.m.s.d.s for bonds (Å)	0.013	0.013
R.m.s.d.s for angles (°)	1.211	1.306
Average *B* value (Å^2^)	39.8	34.9
Ramachandran plot statistics (%)		
Favored	96.3	97.6
Allowed	1.3	2.4
Disallowed	0	0
Rotamer outliers	0.93	1

## Appendix A Uncertainty estimation

We estimate the uncertainties of the integrated intensities from the measurement *K_ik_* by error propagation. Let vector **y** be a set of functions of vector **x**. Their covariance matrices, Λ_**y**_ and Λ_**x**_, can be related by the formula of error propagation,

where *J* denotes the Jacobian matrix of **y**. When **x** and **y** are related by an implicit function, *f*(**x**, **y**) = 0, the Jacobian matrix is given by

From equation (7), the implicit function that relates  and *K_ik_* is

the derivative of the function to be minimized with respect to . Since  is a scalar in equation (13), the Jacobian matrix of  becomes a row vector with length *N*_data_, the number of data frames, and its *k*th element is given by

The covariance matrix of the measurement, , is a diagonal matrix of size *N*_data_ × *N*_data_, with the diagonal terms being the photon counts *K_ik_* as a result of Poisson statistics. Substituting these matrices into equation (11), we obtain the variance of , denoted .

The values of interest are the uncertainties of the integrated intensities, *I_hkl_* = , where {**p**_*hkl*_} represents the three-dimensional grid points within the predefined radius *r*_p_ for the Bragg peak labeled by indices *hkl*. From equation (9), the variance of  is given by

Here, we assume that the tomogram values  contributing to the same Bragg peak are independent variables. This assumption is based on the observation that each data frame only has non-negligible probabilities at a few orientations on convergence, so the values of  with different indices are mostly sampled by different data frames. For the same reason, we also assume that the values *W*(**p**) for **p**, even sampling the same Bragg peak, are independent variables. The variance of *I_hkl_* is hence given by

## Appendix B Computational details

The reconstruction was performed on an Amazon Elastic Compute Cloud (EC2) instance r4.16xlarge, which has 64 virtual CPUs and 488 GB memory. The low-resolution reconstruction used 120 574 data frames with a resolution cutoff of 4 Å, which give a data size of 570 GB. The high-resolution reconstruction used 117 904 selected single-crystal frames with a resolution of up to 2 Å, which give a data size of 2.3 TB. The low-resolution reconstruction, high-resolution reconstruction and calculation of CC* took 41, 25 and 68 h, respectively.
